# Mouldy feed, mycotoxins and Shiga toxin - producing *Escherichia coli *colonization associated with Jejunal Hemorrhage Syndrome in beef cattle

**DOI:** 10.1186/1746-6148-7-24

**Published:** 2011-06-03

**Authors:** Danica Baines, Stephanie Erb, Kelly Turkington, Gretchen Kuldau, Jean Juba, Luke Masson, Alberto Mazza, Ray Roberts

**Affiliations:** 1 Lethbridge Research Centre, 5403 1 Avenue South, Lethbridge, AB, T1J 4B1, Canada; 2 Lacombe Research Centre, 6000 C and E Trail, Lacombe, AB, T4L 1W1, Canada; 3 Penn State, 321 Buckhout Laboratory, University Park, PA, 16802-4508, USA; 4 Penn State, Fusarium Research Center, 216 Buckhout Laboratory, University Park, PA, 16802, USA; 5 Biotechnology Research Institute, National Research Council of Canada, Montréal, QC, H4P 2R2, Canada; 6 Lethbridge Animal Clinic, 3333 1 Avenue South, Lethbridge, AB, T1J 4H1, Canada

## Abstract

**Background:**

Both O157 and non-O157 Shiga toxin - producing *Escherichia coli *(STECs) cause serious human disease outbreaks through the consumption of contaminated foods. Cattle are considered the main reservoir but it is unclear how STECs affect mature animals. Neonatal calves are the susceptible age class for STEC infections causing severe enteritis. In an earlier study, we determined that mycotoxins and STECs were part of the disease complex for dairy cattle with Jejunal Hemorrhage Syndrome (JHS). For STECs to play a role in the development of JHS, we hypothesized that STEC colonization should also be evident in beef cattle with JHS. Aggressive medical and surgical therapies are effective for JHS, but rely on early recognition of clinical signs for optimal outcomes suggesting that novel approaches must be developed for managing this disease. The main objective of this study was to confirm that mouldy feeds, mycotoxins and STEC colonization were associated with the development of JHS in beef cattle.

**Results:**

Beef cattle developed JHS after consuming feed containing several types of mycotoxigenic fungi including *Fusarium poae, F. verticillioides, F. sporotrichioides, Penicillium roqueforti *and *Aspergillus fumigatus*. Mixtures of STECs colonized the mucosa in the hemorrhaged tissues of the cattle and no other pathogen was identified. The STECs expressed Stx1 and Stx2, but more significantly, Stxs were also present in the blood collected from the lumen of the hemorrhaged jejunum. Feed extracts containing mycotoxins were toxic to enterocytes and 0.1% of a prebiotic, Celmanax Trademark, removed the cytotoxicity *in vitro*. The inclusion of a prebiotic in the care program for symptomatic beef calves was associated with 69% recovery.

**Conclusions:**

The current study confirmed that STECs and mycotoxins are part of the disease complex for JHS in beef cattle. Mycotoxigenic fungi are only relevant in that they produce the mycotoxins deposited in the feed. A prebiotic, Celmanax Trademark, acted as a mycotoxin binder *in vitro *and interfered with the progression of disease.

## Background

Adult cattle are the main reservoir for O157 and non-O157 STECs, bacteria that cause serious human disease outbreaks resulting in symptoms that include hemorrhagic colitis or hemolytic uremic syndrome. Until recently, there have been no reports of O157 STEC disease in mature cattle [1], but STECs do affect calf health from birth to weaning [2-4]. STEC infections cause high mortality in neonatal calves resulting from acute enteritis [5,6]. Older calves have transient watery diarrhea but are not seriously affected by O157 STEC infections, while this age class is susceptible to non-O157 STEC infections [2-4]. The characteristic patchy attachment/effacement (A/E) lesions are always present in hemorrhaged tissues of humans [7]. These lesions are similarly found in the jejunum, ileum, cecum, colon, and rectum in neonatal calves, but not in older calves [5,6,8,9]. The O157 STEC can adhere to and form A/E lesions in intestinal tissue from mature cattle *in vitro *and *in vivo *[1,7,10-12]. If STECs do cause disease in mature cattle, the most likely candidates are diseases with unclear etiologies such as JHS [13]. Current treatments for JHS include an aggressive medical and surgical therapy that can be effective, but the prognosis for long term survival relies upon early detection [14]. An alternative therapy for on-farm treatment is required for animals that are not detected or for rapidly developing cases. In monogastric livestock, prebiotic feed additives favor the growth of beneficial bacteria in the intestinal tract, remove pathogenic bacteria from the intestinal tract, increase cytokine levels and increase percentages of lymphocyte subpopulations suggestive of an overall health benefit [15-17]. Very little information is available concerning the benefits of prebiotics for managing pathogens in beef cattle. The first objective of the current study was to characterize pathogen colonization in hemorrhaged jejunum of beef cattle during natural JHS outbreaks. The second objective was to confirm the association of mycotoxigenic fungi in feeds with the development of JHS and also to identify the presence of mycotoxins. The third objective was to determine the impact of a prebiotic, Celmanax™, on the feed-associated mycotoxin cytotoxicity *in vitro*. The final objective was to determine if a prebiotic could modify the progression of disease in beef cattle.

## Results

### Pathology and impact of a prebiotic on animal health

The six samples of jejunum obtained from Angus-cross beef cattle at a local abattoir had no pathology and no STEC detected in the tissue. The single application of Celmanax™ was associated with a rapid recovery in 48 hr for 11 of the 16 symptomatic beef feeder calves on BF1. The recovered calves had no further health issues. Five calves succumbed to the disease, but these calves had hindlimb paralysis within 24 hr of consuming the mouldy feed and did not regain their mobility. Necropsy of the calves from BF1 revealed the presence of hemorrhaged tissue about 1 meter long in the jejunum for all five calves. There were also raised Peyer's patches, severe focal hemorrhages, bloody digesta, mucosal erosions, dark-red erythema, large blood clots and edema. The hemorrhages and blood-filled distended loops were visible through the serosa. In contrast, necropsy of finishing beef cattle from BF2 revealed the same pathology but with smaller blood clots than observed in BF1 samples. The severe hemorrhaging and distended loops of blood were not visible through the serosa, perhaps because of the severity of edema. The pathology identified in the jejunal samples from BF1 and BF2 confirm the JHS diagnosis.

### STEC isolation from the jejunum of JHS cases

Adherent non-sorbitol fermenting colonies were identified at significant levels colonizing the hemorrhaged regions of the jejunum from BF1 relative to the control animals (*P *= 0.001, Table 1). There was about a 10 fold higher STEC colonization of the mucosal tissue in the finishing cattle (7.78 ± 0.32 Log CFU/2.5 cm^2 ^tissue) compared with the beef feeder calves (6.49 ± 0.39 Log CFU/2.5 cm^2 ^tissue). Similarly, there was 10 fold higher STEC in the bloody digesta for the finishing cattle (6.22 ± 0.02 Log CFU/ml) compared with the beef feeder calves (5.55 ± 0.18 Log CFU/ml). There were also sorbitol-fermenting colonies that represented about 50% of the CFU/sample/site but we did not initially suspect them as non-O157 STECs. The non-sorbitol fermenting bacterial isolates were confirmed as O157 and H7 using the RIM™ E. coli O157:H7 Latex test. The sorbitol fermenting colonies weakly agglutinated using the RIM™ E. coli O157:H7 Latex test. The STEC isolates were examined for the expression of Stx, as this is a key virulence trait associated with more severe forms of animal disease. The isolates produced Stx1 and Stx2. The consistent presence of significant numbers of STEC in the hemorrhaged regions for cattle with JHS regardless of the feedlot, suggests that the STECs warrant further investigation as causative agents.

**Table 1:** Average Shiga toxin - producing *Escherichia coli *Log CFU / sample type for beef cattle with or without JHS

Sample type	BF1 Cattle with JHS (n = 5) Mean Log CFU ± SE	Cattle without JHS (n = 6) Mean Log CFU ± SE
**Jejunum (per 2.5 cm^2 ^mucosa)^1^**	6.49 ± 0.39a	0.000 ± 0.000b

**Digesta (per ml)**	5.55 ± 0.18a	0.000 ± 0.000b

**1 row numbers followed by different letters are significantly different *P *= 0.001**.

### Isolation of bacterial pathogens from the jejunum of JHS cases

CHROMagar™ is a selective media used for presumptive identification of specific pathogens including *Salmonella, E. coli, E. coli *O157 and *Listeria *[18-20]. Presumptive *E. coli *O157 were identified and appeared as regular mauve colonies (O157 STEC and non-O157 STEC) or navy blue colonies with a mauve halo (non-O157 STEC) on CHROMagar™ O157 plates. Presumptive *E. coli *O157 were confirmed as pathogenic *E. coli *in the GN-ID A + B test. Other presumptive pathogenic *E. coli *appeared as blue colonies on CHROMagar™ E. coli plates. All *E. coli *were confirmed as non-pathogenic *E. coli *in the GN-ID A + B test. White colonies were also found on this plate that were mauve on CHROMagar™ O157 plates and were identified as pathogenic *E. coli *in the GN-ID A + B test. This provided a crosscheck for the results from the CHROMagar™ O157 plates and for the non-sorbitol or sorbitol-fermenting colonies. Presumptive *Salmonella *were identified and appeared as mauve colonies on CHROMagar™ Salmonella Plus plates, but were subsequently determined as false positives (pathogenic *E. coli *using the GN-ID A+ B test and the Salmonella Latex Agglutination test). No *Listeria *species were identified. No *Clostridium perfringens *was detected. Thus, the only bacterial pathogen identified in the hemorrhaged tissues or bloody digesta for beef cattle with JHS regardless of the feedlot, were STECs.

### DNA microarray assay targeting *E. coli *genes

The results from the bacterial isolations from the SMAC, CT-SMAC and CHROMagar™ O157 plates suggested that the tissues had at least two types of STEC present, O157 and non-O157. There were two distinct morphologies on the CHROMagar™ O157 plates: mauve colonies with a circular form, raised elevation and entire margin; blue colonies with a mauve halo that had a circular form, raised elevation and an entire margin. These colonies were non-sorbitol and sorbitol fermenting with both types of STECs tellurite-resistant. The DNA microarray assay confirmed the presence of the non-O157 STEC in the hemorrhaged tissues and these isolates have the locus of enterocyte effacement (LEE) pathogenicity island, *stx1A, stx1B *and *hlyA*. Finally, in addition to the genes mentioned above, variability in the presence of other virulence genes also supports the notion of a mixed STEC population.

Interestingly, the absence of the *stx2 *gene in the characterized isolates suggests that the detected Stx2 from the isolate and blood samples may represent a strain that was not ultimately isolated or the loss of genes from sub-culturing procedures [21].

### Mycotoxigenic fungi in silages and tissues

There were no visible hyphae (400 times magnification; Nikon Diaphot inverted microscope) or fungal growth from tissues of the beef calves or finishing beef cattle. *Fusarium *species were present in the digesta of the hemorrhaged regions from the cattle with JHS. There was no *Aspergillus fumigatus *present in the feed samples from BF1, but it was present in the barley silage from BF2. There were several other types of mycotoxigenic fungi present in the barley silage from BF1 including *F. verticillioides, F. sporotrichioides *and *P. roqueforti *(Table 2). The hay from BF1 also had mycotoxigenic fungi present, *F. poae *and *P. roqueforti*. No mycotoxigenic fungi were detected in other feed components from BF1. The barley silage from BF2 had two mycotoxigenic fungi present, *F. verticillioides *and *P. roqueforti *(Table 2).

**Table 2:** Percent of samples positive for mycotoxigenic fungi in barley silage and hay from beef feedlots (n = 5-10)

Mycotoxigenic Fungi	BF1 Barley Silage	BF2 Barley Silage	BF1 Hay
***Fusarium poae***	0	0	100

***Fusarium verticillioides***	100	100	0

***Fusarium sporotrichioides***	100	0	0

***Aspergillus fumigatus***	0	100	0

***Penicillium roqueforti***	100	100	100

### Lawn assay for cytotoxicity associated with feed extracts

**Figure 1:**
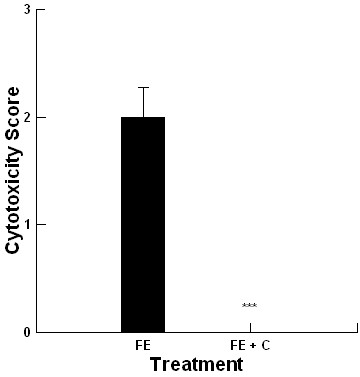
Impact of 0.1% Celmanax™ (C) on the cytotoxicity of feed extracts (FE) (n = 6; ***, *P = 0.001*).

For BF1, the extracts from the 2008 barley silage had a higher Cytotoxicity Score (1) compared with the 2007 barley silage that had no activity. Interestingly, the hay extracts from BF1 had a high Cytotoxicity Score (3) equivalent to those reported with similar JHS outbreaks in 5 dairy production sites in southern Alberta [22]. All other feed components from BF1 had no detectable cytotoxicity. The barley silage from BF2 had a lower Cytotoxicity Score (2) compared with the hay in BF1. The Cytotoxicity Scores were pooled for all feed samples having activity and are presented in Figure 1. The inclusion of 0.1% concentration of Celmanax™ removed the cytotoxicity associated with all extracts regardless of the production site (Figure 1, *P *= 0.001).

## Discussion

JHS is a hemorrhagic disease in dairy and beef cattle with the main pathology presenting as hemorrhaging, severe inflammation, mucosal erosion and large blood clots in the jejunum [13]. Initially, two factors, mouldy feed containing *A. fumigatus *and a pathogen, *C. perfringens *type A, were being investigated as causative agents [23-25]. We recently expanded the disease complex to include mycotoxins and STECs based on a study of JHS cases from five dairy production sites [22]. The current study confirmed earlier reports suggesting that *C. perfringens *type A was not involved [23-25]. The pathologies observed in the beef cattle in this study are the same as those reported in other JHS cases [22-25] and as described for O157 STEC infections in experimental challenged beef cattle that were persistent shedders [1]. Similarly, the amount of STEC colonization detected in this study and a previous study with dairy cattle are equivalent to that described for O157 STEC infections in experimental challenged beef cattle that were persistent shedders [1,22]. Together these studies support further investigation of STECs as part of the disease complex for JHS. *Aspergillus fumigatus *was reported to infect tissues in dairy cattle with JHS and it was suggested that the *A. fumigatus *infection deposited mycotoxins in the blood that then contributes to the development of JHS [13]. We did find low levels of *A. fumigatus *in the feed from BF2, but not for BF1 or other production sites where natural JHS outbreaks occurred [22]. In support of a lesser role for the fungi in the disease complex, we also did not confirm tissue infections. Future studies should examine the type and concentration of mycotoxins in feed components together with STEC colonization of hemorrhaged tissues in cattle to confirm their role in JHS.

The dominant fungi found in the hay or silages in this study were *F. poae *which produce T-2 toxins and *F. sporotrichioides *which produce fumonisins [26,27]. Both mycotoxins were identified at significant levels in feed components consumed by dairy cattle that developed JHS and this corresponded to a high Cytotoxicity Score (3) [22]. The co-occurrence of hemorrhaging in the intestinal tract of cattle with the consumption of T-2 or fumonisin contaminated feeds suggests that the mycotoxins could be a factor in the development of JHS. However, experimental challenge studies with T-2 in cattle determined that this material caused severe congestion of the mucosa, congestion of the villar tips and severe congestion of the vasculature in the lamina propria of the jejunum, but no hemorrhaging [27]. Similarly, fumonisin B1 is cytotoxic to epithelial cells, inhibits immune function, promotes a loss in mucosal barrier function, causes diarrhea and abdominal pain, but does not cause hemorrhaging [26]. This suggests that the mycotoxins could have a role in promoting pathogen colonization in cattle by providing colonization niches, toxin entry into the submucosa or blood stream and preventing early recognition of an infection. In human infections, early non-intimate STEC colonization is important and clinically is characterized by non-bloody diarrhea. An odd diarrhea was a common symptom reported by the producers at the beef production sites in this study and in earlier dairy cattle studies [22]. In both studies, the consistent co-occurrence of high Cytotoxicity Scores or "mycotoxin content" in one or more feed components supports a role for mycotoxins in the disease complex for JHS. As STEC colonization progresses in human infections, intimate-attachment and Stx production contribute to the development of severe hemorrhaging. Cattle do not have the appropriate receptors to facilitate Stx entry, but it is possible that the types of damage reported for the mucosal exposure to mycotoxins such as T-2 or fumonisin could provide the mechanism for toxin activities [28]. Future studies should examine the interaction of specific mycotoxins and STEC in cattle to determine if these factors elicit disease.

Antibiotic treatment for STEC infections in humans does not change STEC colonization or development of more serious forms of disease. More importantly, the administration of some types of antibiotics such as trimethoprim, increases the expression of Stxs resulting in faster development of disease [29]. A common comment from producers that have experienced JHS outbreaks is that antibiotics were ineffective and if anything the clinical symptoms worsened after treatment [22]. In southern Alberta, Trivetrin that contains trimethoprim is commonly used to treat cattle ailments such as diarrhea suggesting that the administration of this antibiotic early in the infection process may have contributed to the development of JHS, but also to STEC carriage. The novel prebiotic treatment used in this study resulted in 69% recovery of the symptomatic beef calves. Since the prebiotic acts as an anti-adhesive for STEC colonization and a mycotoxin binder *in vitro*, the results suggest that the benefit to the calves could have been derived from preventing mycotoxin:STEC interactions [22]. Future studies should determine whether prebiotic treatments could be used to manage JHS cases.

## Conclusion

The current study confirmed that mycotoxins and STEC are part of the disease complex for JHS in beef cattle. We did not confirm a role for *C. perfringens *type A or mycotoxigenic fungi in JHS. A prebiotic treatment alleviated the development of disease in symptomatic beef calves. Future studies should examine the role of STECs and mycotoxins in the infection process that leads to JHS and the mode of action of prebiotics.

## Methods

### Beef feedlots and impact of a prebiotic on animal health

The Animal Care Committee reviewed the study and concluded that the observational research on commercial production sites did not require authorization. As such, animal care followed the code of practice for farm animals described in the guidelines for beef cattle (National Farm Animal Care Council of Canada). The producer granted permission for the post-mortems on beef calves or finishing cattle succumbing to disease and to monitor the impact of the feed additive on animal health. A shipment of five hundred beef feeder calves (227 kg) was received by a commercial beef feedlot (BF1) in Southern Alberta in December 2008. The animals appeared normal with no evidence of illness in the first two weeks. The producer purchased hay and began feeding it to the calves with no apparent problem until the next day. Fifty beef-feeder calves were dead in the morning with the only visible symptoms melena or black, "tarry" feces that are associated with gastrointestinal hemorrhage together with frank blood in the feces. Thirty-four calves were transferred to a sick pen with 16 animals seriously affected. The producer believed that the hay was responsible and removed it. Eighteen animals recovered over 7 days, the remaining 16 calves continued to decline even after antibiotic treatments were applied. The clinical symptoms suggested a mycotoxicosis including staggering, paralysis and wasting. The producer tried an alternative approach, a prebiotic feed additive, to reverse the wasting or to assist digestion. The prebiotic, Celmanax™ (VI-COR^®^, Mason City, IA, USA), consists of a non-living formulation of yeast cell walls or mannan-oligosaccharide (MOS) and improves cattle performance [30]. The producer administered a single 400 ml drench of liquid Celmanax™ to all sixteen calves. We monitored the 16 calves to determine if they improved or continued to decline.

Another beef feedlot (BF2) for finishing cattle (9000 animals) in Southern Alberta experienced sudden onset death in October, 2010. Twelve cattle died overnight and after several postmortems, no apparent cause was found. The next day, two additional cattle died with no symptoms or apparent cause. After discussions with the veterinarian, a sample was removed from the mid-region of the jejunum, where we have had success in the past in identifying hemorrhaged tissue regardless of the lack of any visible hemorrhaging through the serosa.

### Pathology and STEC isolation from the jejunum of JHS cases

Six beef cattle were chosen randomly from an abattoir in Southern Alberta and samples were collected from the mid-section of the jejunum. These tissues served as negative controls. The tissue from 5 beef cattle on BF1 confirmed a JHS diagnosis based on the presence of acute hemorrhaging in the jejunum, blood in the small intestine and black, "tarry" feces. To compare pathology associated with JHS diagnosis at BF1 and BF2, tissue samples were collected and the pathology recorded as described previously [22]. Briefly, a 30 cm piece of tissue was removed from the acute hemorrhaged region of the jejunum. The blood and tissue were separated into sample tubes before serial dilutions were plated on Sorbitol MacConkey agar (SMAC; Dalynn Biologicals, Calgary, Alberta, Canada) to identify non-sorbitol fermenting bacterial colonies and on Potato Dextrose agar (PDA; Dalynn Biologicals, Calgary, Alberta, Canada) to isolate fungi. Adherent or tissue-colonizing bacteria were collected by incubating a 2.5 cm^2 ^tissue piece in 0.1% Triton X-100 at 4°C overnight and plated on SMAC agar to determine the presence of non-sorbitol fermenting bacterial colonies. Any suspect colonies were tested as O157 and H7 using the RIM™ E. coli O157:H7 Latex test (Fisher Scientific, Ottawa, Ontario, Canada).

To elucidate whether STEC virulence determinants were critical to the development of JHS, the blood in the hemorrhaged jejunum from BF2 and the presumptive *E. coli *O157:H7 isolates from the hemorrhaged regions were examined for Stx1 and Stx2 expression using the Meridian™ ImmunoCard STAT!^® ^EHEC test (Somagen, Edmonton, Alberta, Canada).

### Isolation of bacterial pathogens from the jejunum of JHS cases

Washed tissue samples (2.5 cm^2^) were placed in 0.1% Triton X-100 overnight and the released bacteria stored at -80°C or serial dilutions were direct plated. The digesta was also stored at -80°C or serial dilutions were direct plated. A 1 to 50 μl aliquot of a diluted sample was applied to CHROMagar™ Salmonella, CHROMagar™ E. coli, CHROMagar™ O157, CHROMagar™ Salmonella Plus, and CHROMagar™ Listeria plates (Dalynn Biologicals, Calgary, Alberta). To confirm identity, the presumptive isolates with the exception of the *Listeria *were subjected to a Microgen™ GN-ID A + B biochemical test (Alere™ Canada, Ottawa, Ontario). Presumptive *Salmonella *were also subjected to a Microgen™ Salmonella Latex Agglutination test (Alere™ Canada, Ottawa, Ontario, Canada). Presumptive *Listeria *was identified using a Microgen™ Listeria ID test (Alere™ Canada, Ottawa, Ontario). To detect *C. perfringens *type A, samples were examined for characteristic features using a compound Nikon microscope set at 1000× magnification. Digesta and tissue smears were examined for the presence of large, rectangular bacilli (rod) with or without spores (ovoid, sub-terminal). Sub-cultures on blood agar were examined for rapid spreading growth.

### DNA microarray assay targeting pathogenic *E. coli *genes

The original samples were re-examined for multiple STECs as described previously [22]. The microarray (MaxiVir1.0) used in this study was based on earlier published work and carries 514 oligonucleotides of 70 bases in length targeting 348 virulence or virulence-related genes and 96 antimicrobial resistance or antimicrobial resistance-related genes found in gram-negative bacteria [31]. The microarray, designed to detect a complete set of virulence genes representative of all *E. coli *pathotypes, includes virulence factors such as adhesins, locus of enterocyte effacement, colicins and microcins, toxins, iron acquisition and transport systems, capsular and somatic antigens, hemolysins and hemaglutinins, as well as newly recognized or putative *E. coli *virulence genes. Antimicrobial resistance genes included in the microarray represent different antimicrobial families such as ß-lactams, aminoglycosides, tetracycline, phenicols, trimethoprim, sulfonamide and class I integron. The microarray also carries five positive oligonucleotide controls for *E. coli *derived from the sequences of genes encoding tryptophanase (*tnaA*), beta-glucuronidase (*uidA*), lactose permease (*lacY*), beta-galactosidase (*lacZ*), and glutamate decarboxylase (*gad*). Negative controls added to this microarray consist of oligonucleotides derived from the gene sequences for the green fluorescent protein of *Aequorea victoria*, the lactose permease of *Citrobacter freundii*, and the chlorophyll synthase from *Arabidopsis thaliana*.

### *Escherichia coli *DNA labeling

Bacterial DNA was labeled using Bioprime DNA labeling system (Invitrogen Life Technologies, Burlington, ON, Canada). Fifteen μl of the supernatant containing DNA was added to a final volume of 32.5 μl containing 10 μl of a random primer solution, 0.5 μl of high-concentration DNA polymerase (Klenow fragment, 40 U/μl), 5 μl of a deoxyribonucleosidetriphosphate (dNTP) mixture (1.2 mM dATP, 1.2 mM dGTP, 1.2 mM dTTP, and 0.6 mM dCTP in 10 mM Tris [pH 8.0] and 1 mM EDTA), and 2 μl of 1 mMCy5-dCTP. Labeling reactions were performed in the dark at 37°C for 3.5 h and stopped by the addition of 5 μl Na_2_EDTA 0.5 M (pH 8.0). The labeled samples were then purified with a PureLink PCR purification kit (Invitrogen Life Technologies, Carlsbad, CA) according to the manufacturer's protocol. The amount of incorporated fluorescent Cy5 dye was then quantified by scanning the DNA sample with a NanoDrop ND-1000 spectrophotometer from 200 to 700 nm. Data were analyzed using a Web-based percent incorporation calculator http://www.pangloss.com/seidel/Protocols/percent_inc.html.

### Hybridization of labeled DNA

Microarrays were prehybridized at 50°C for 1 hour under a Lifterslip (25 × 60 mm; Erie Scientific Company, Portsmouth, NH, USA) using a SlideBooster hybridization workstation (model SB800; Advalytix, Germany), with 50 μl of prewarmed (37°C) digoxigenin (DIG) Easy Hyb Buffer (Roche Diagnostics, Laval, Quebec, Canada) supplemented with 5% (vol/vol) bovine serum albumin (1 mg/ml; New England Biolabs Inc., Beverly, MA). After pre-hybridization, the lifterslip was removed by dipping the slides in 0.1X SSC (saline-sodium citrate) and were air-dried. Before hybridization, the samples were dried and resuspended in 15 μl of hybridization buffer (DIG + 0.1 ug/ul ssDNA) and denatured for five minutes at 95°C. One microgram of labeled genomic DNA was hybridized on the MaxiVir1.0 microarray under a lifterslip (18 × 18 mm). The hybridization was carried out overnight at 50°C in a SlideBooster hybridization workstation. After hybridization, lifterslips were removed by dipping the slides in a 0.1X SSC and 0.1% SDS (sodium dodecyl sulfate) solution. Post-hybridization washes were performed at 37°C: two washes with 0.1X SSC and 0.1% SDS for ten and five minutes respectively and one last wash in 0.1X SSC for five minutes. The microarrays were then air-dried.

Microarray slides were scanned at 5 μm resolution with a ScanArray Lite fluorescent microarray analysis system (Perkin-Elmer, Missasauga, Ontario, Canada). Acquisition of fluorescent spots was performed using the ScanArray Express software (Perkin-Elmer, Foster City, CA). Fluorescent spot intensities were quantified with ImaGene software version 8.0 (BioDiscovery, Inc., El Segundo, CA). All the microarrays were normalized using the same method. For each subarray, the mean value for each set of duplicate spotted oligonucleotides was divided by the correction factor taken from the negative controls spots. This value was then divided by the average of the empty spots to create a signal-to-noise ratio. Oligonucleotide spots with a signal-to-noise fluorescence ratio greater than the established threshold (3 in this case), were considered positive. These ratios were then converted into binary data where a value of 0 indicates a negative probe and a value of 1 a positive probe. A threshold of 3 was chosen because it best represented spot quantification. To verify that the results were accurate, we compared the.bmp image of a given sample and the quantified result.

### Isolation and identification of mycotoxigenic fungi

Feed components were collected from BF1 and the barley silage was collected from BF2. A 10 g sub-sample was finely ground and a 5 ml volume added to a PDA plate. The plate was incubated for 1 to 7 days and individual fungal isolates transferred to new PDA plates. Tissue smears were examined for fungal hyphae and suspect tissue was placed on a PDA plate. *Fusarium *isolates were identified by examination of micro-morphological characters and by PCR amplification and sequencing of a fragment of the *EF1-a *gene and comparing the sequence with the FUSARIUM-ID database [32,33]. *Penicillium *isolates were identified by microscopic examination of morphology using the guide by Pitt [34]. *Aspergillus *isolates were identified by microscopic examination of morphology using the guide provided by Klich [35].

### Extraction of cytotoxins from feed components

The methods used have been described previously [36]. The feed samples were not visibly mouldy for BF1 or BF2. The components of the total mixed ration and hay were collected from BF1 while the barley silage was collected from BF2. To extract the mycotoxins, each sample was finely ground, a 25 ml aliquot of 50% methanol was added to a 3 g sample and placed on a shaker at 200 rpm for 3 h. The supernatant was collected in another tube, and stored at 4°C until use.

### Lawn assay for cytotoxicity associated with feed extracts

The lawn assay has been described previously and was used to examine the cytotoxicity of the toxins produced by *Escherichia coli *O157:H7 strains [37] and to detect mycotoxins in feeds [22]. The assay was performed using the feed extracts in the absence or presence of 0.1% Celmanax™. Briefly, a 1% SeaKem^® ^agarose (Mandel Scientific, Guelph, Ontario, Canada) support gel was poured into a petri dish. Next, the lawn agarose [3 ml of 3.7% SeaPlaque^® ^agarose (Mandel Scientific, Guelph, ON, Canada)] was mixed with 3 ml of enterocyte suspension or a bovine colonic cell line and poured over the support agarose. A 5 ul aliquot of the solvent used for the extraction process served as negative controls. Each extract (5 μl) was applied with or without 0.1% prebiotic and the plate incubated for 4 h under standard culture conditions. The lawn was stained with 0.1% trypan blue (Sigma-Aldrich) and de-stained using PBS. Plates were scored the same day and the amount of extract cytotoxicity was scored as low (Cytotoxicity Score 1), moderate (Cytotoxicity Score 2) or high (Cytotoxicity Score 3) which was visualized as a faint blue spot, a blue spot or a dark blue spot respectively. These activities were compared to two standards, ground corn containing 0.1 ppm aflatoxin that had a low Cytotoxicity Score (1) and 1 ppm aflatoxin that had a high Cytotoxicity Score (3). The assay was repeated a minimum of three times.

### Data analysis

All data were analyzed using ANOVA followed by a posthoc Tukey's test for comparison of the means. Results were considered significant if *P *< 0.05 and non-significant if *P *> 0.05.

## Competing interests

The authors declare that they have no competing interests.

## Authors' contributions

DB conceived of the study, designed the study, collected tissue, performed the mucosal studies, isolated fungi, extracted feed components, performed the lawn assay, performed the statistical analysis and drafted the manuscript; SE isolated fungi, prepared tissues, extracted feeds, performed lawn assays; KT provided morphological identification of the fungi; GK provided morphological and PCR identification of fungi; JJ performed the PCR bioassays for fungal identification; LM conceived of the study to characterize the O157 and non-O157 STEC; AM performed the DNA microarrays for STEC identification; RR provided tissue samples, consulted on pathology and disease diagnosis. All authors read and approved the final manuscript.
